# Viewing Loved Faces Inhibits Defense Reactions: A Health-Promotion Mechanism?

**DOI:** 10.1371/journal.pone.0041631

**Published:** 2012-07-23

**Authors:** Pedro Guerra, Alicia Sánchez-Adam, Lourdes Anllo-Vento, Isabel Ramírez, Jaime Vila

**Affiliations:** 1 Department of Personality, University of Granada, Granada, Spain; 2 Department of Personality, Spanish National Open University, Madrid, Spain; 3 F. Olóriz Institute of Neurosciences, University of Granada, Granada, Spain; University of Bologna, Italy

## Abstract

We have known for decades that social support is associated with positive health outcomes. And yet, the neurophysiological mechanisms underlying this association remain poorly understood. The link between social support and positive health outcomes is likely to depend on the neurophysiological regulatory mechanisms underlying reward and defensive reactions. The present study examines the hypothesis that emotional social support (love) provides safety cues that activate the appetitive reward system and simultaneously inhibit defense reactions. Using the startle probe paradigm, 54 undergraduate students (24 men) viewed black and white photographs of loved (romantic partner, father, mother, and best friend), neutral (unknown), and unpleasant (mutilated) faces. Eye–blink startle, zygomatic major activity, heart rate, and skin conductance responses to the faces, together with subjective ratings of valence, arousal, and dominance, were obtained. Viewing loved faces induced a marked inhibition of the eye-blink startle response accompanied by a pattern of zygomatic, heart rate, skin conductance, and subjective changes indicative of an intense positive emotional response. Effects were similar for men and women, but the startle inhibition and the zygomatic response were larger in female participants. A comparison between the faces of the romantic partner and the parent who shares the partner’s gender further suggests that this effect is not attributable to familiarity or arousal. We conclude that this inhibitory capacity may contribute to the health benefits associated with social support.

## Introduction

Over the last several decades, evidence has accumulated on the fundamental role that social factors play in brain organization and function [1], [2], as well as in preserving physical and mental health [3], [4]. For humans, survival depends on effective social functioning. Care giving and attachment, the two key elements of love [5], are essential not only for survival during infancy and childhood, but also for physical and psychological wellbeing across the life span [6]. Social support, defined as receiving information that one is loved, valued, and part of a social network, has been known for decades to be associated with reduced morbidity and mortality rates [6], [7]. Recent neuroscience research on social support has examined the effects of both positive and negative aspects of social environments on genetic expression, physiological functioning, and brain activity.

Regarding genetic expression, it has been reported, for instance, that a loving and caring family reverses the expected negative effects of the short/short polymorphism in the serotonin transporter gene (5-HTTLPR), which is associated with depression and other forms of psychopathology [8]. In terms of physiological functioning, it has been found that the presence of a loved person during the preparation for an acute stress task (the *Trier Social Stress Test*) reduces activity in the hypothalamic pituitary-adrenal axis (cortisol) during the task [9]. This effect was enhanced by intranasal oxytocin administration, the neuropeptide implicated in both parent-child bonding and prosocial behaviors [10]. With regard to brain activity, the conditions of perceived social isolation [11] and social exclusion [12], characterized by lack of support have been found to be associated with both reduced activation in brain reward areas and increased activation in areas involved in the defense system.

The link between social support and positive health outcomes is likely to depend on the neurophysiological regulatory mechanisms underlying reward and defense reactions. Some researchers have argued [12] that unsupportive social environments, especially those that lead to social exclusion, play the role of threatening cues that activate both the defense motivational system and the broad spectrum of stress responses known to adversely impact an organism’s physical and mental health [13]. Loving environments may play the opposite role, providing safety cues that simultaneously activate the appetitive reward system and inhibit defense reactions. To support this idea, a recent study [14] has shown that female participants rated painful stimulation as less painful when viewing the picture of a romantic partner, compared to the picture of a stranger. They also displayed reduced neural activation in pain-related regions and increased activation in safety signal-related regions. However, to date no study has directly examined the capacity of loved faces for inhibiting defense reactions. Here, we set out to confirm this hypothesis by means of the startle probe paradigm.

The startle probe paradigm is one of the strongest and most recent paradigms developed to study the neurophysiological mechanisms of appetitive and defense reactions, as well as their reciprocal inhibitory function [15]. In this paradigm, the modulation of the eye-blink startle reflex elicited by a noise burst, together with other peripheral (heart rate, skin conductance, zygomatic major muscle activity, and corrugator supercilii muscle activity) and central (event-related potentials) physiological responses, is examined while participants view pleasant, neutral, and unpleasant pictures selected from the *International Affective Picture System* (IAPS). Using this paradigm, Lang and his colleagues [15] have consistently demonstrated that the magnitude of the eye-blink response to the noise burst is augmented when people are viewing highly unpleasant pictures and diminished when viewing highly pleasant ones. They explain this modulation as due to the congruence versus incongruence between the motivational system engaged by the perceptual stimuli and the type of reflex that is being elicited (*motivational priming hypothesis*). Thus, unpleasant stimuli that engage the defense motivational system potentiate defense reflexes, while pleasant stimuli that engage the appetitive motivational system inhibit those same defense reflexes.

The startle probe paradigm has never been used to investigate the neurophysiological mechanisms of love. This paradigm has two basic elements: the passive viewing of pictures and the elicitation of the eye-blink startle reflex. Interestingly, a number of recent studies have used a modification of the picture viewing procedure by substituting pleasant pictures with photographs of loved, familiar faces [16]–[24]. However, none of these studies recorded eye-blink startle or other peripheral physiological measures that might confirm elicitation of a genuine positive emotional response to the faces. Almost all these studies restricted the physiological measures to central indices of brain activity (ERP and fMRI). A major problem in these studies is the absence of clear evidence concerning elicitation of such a positive emotional response. Two confounding factors are always merged in emotional studies that exclusively use central physiological measures: emotional arousal and familiarity. Emotional arousal refers to the intensity of an emotion, regardless of its affective valence (whether positive or negative). The same electrophysiological brain responses to loved familiar faces (i.e., larger P3 and Late Positivity Potentials) have been found in response to highly unpleasant pictures [25], [26], thus calling into question whether the larger ERPs evoked by loved faces are indicative of positive emotional mechanisms or simply reflect undifferentiated emotional arousal.

Familiarity refers to a form of explicit or declarative memory [27]. This type of memory involves the ability to recollect events and factual knowledge about a person, which depends on many factors, including length of time spent with the person. Studies on explicit facial memory [28], [29] have consistently reported that larger P3 and Late Positive Potential amplitudes at posterior locations are associated with familiarity. Attempts to control for familiarity in studies on loved familiar faces include viewing faces of acquaintances, famous people, or newly learned faces. But the familiarity of loved familiar people will always exceed that of control faces because of the greater amount of time spent with them [23]. Thus, the most consistent finding in terms of cortical brain potentials to loved familiar faces –i.e., larger P3 amplitudes and Late Positive Potentials- cannot be attributed to positive emotional responses because similar enhanced brain responses are consistently associated with both undifferentiated emotional arousal and explicit facial memory.

The startle probe paradigm goes beyond elucidating the inhibitory capacity that viewing loved faces has on the startle reflex by including simultaneous recording of peripheral neurophysiological measures, together with subjective reports, that allow unambiguous differentiation between positive emotion, arousal, and familiarity. In addition to reduced startle responses, highly arousing pleasant pictures are associated with a pattern of accelerative changes in heart rate, increases in zygomatic major activity, and decreases in corrugator supercilii activity. The opposite response pattern is associated with highly arousing unpleasant pictures. On the other hand, both highly arousing pleasant and unpleasant pictures are associated with larger skin conductance responses. Using these measures, our group has shown in two recent studies [30], [31] that, when female university students view loved, familiar faces, a marked increase in zygomatic activity and a pattern of heart-rate accelerative changes (indicative of positive emotion [26]), together with an increase in skin conductance (indicative of undifferentiated arousal [26]), is elicited. Additionally, the second study compared two categories of loved faces: one with higher familiarity but lower emotionality (father) and the other with lower familiarity but higher emotionality (romantic partner). Familiarity was defined in terms of amount of time spent with the father and the romantic partner [23]. The results revealed larger responses to the less familiar face, thus suggesting that familiarity is not the key factor in explaining the observed responses.

The aim of the present study was to test the hypothesis that viewing loved familiar faces, compared to neutral (unknown) and unpleasant (mutilated) faces, inhibits the eye blink startle reflex. The study also intended to replicate the previous findings on women and extend them to men. Participants were required to have a romantic partner and a satisfactory relationship with their partner, father, mother, and best friend (opposite sex from partner). As in the second study [31], they were also required to have lived with their parents until they were at least 18 years old, whereas their relationship with the romantic partner could not exceed 6 years. Control faces were neutral faces (four faces selected from the loved-faces category provided by other participants) and unpleasant faces (four mutilated faces taken from the *International Affective Picture System* [32]). To control for familiarity, two loved faces were also compared: the face of the romantic partner (lower familiarity) and the face of the parent of same gender as partner (higher familiarity).

## Methods

### Ethics Statement

The research was approved by the Ethical Committee of the University of Granada (Spain) and was conducted according to the Declaration of Helsinki. All subjects signed written informed consent forms and received course credits for their participation.

### Participants

Participants were 54 healthy undergraduate students (24 of whom were men). All were right-handed and had normal or corrected-to-normal vision. Before the physiological session, participants completed a set of questionnaires to assess general health [33], social support [34], empathy [35], attachment [36], and positive-negative affect [37]. They also rated the familiarity and quality of their relationship with the romantic partner and their parents using a rating scale from 0 to 100. As expected, the familiarity was higher for the parents than for the romantic partner, whereas the quality of their relationship was highly positive in both cases. Results concerning the other questionnaires are being reported separately.

### Stimuli and Task

Four faces of loved people (father, mother, romantic partner, and best friend), four faces of unknown people (selected from the loved faces provided by other participants), and four unpleasant faces (mutilated faces*)* were used. Pictures of loved faces were provided by the participants following specific instructions (i.e., the pictures should not be taken by the participants themselves, and the photographed people were required to look straight at the camera with a neutral expression). Photographs were edited and matched for size, color (black and white), and background. The pictures were presented on a 19″ flat screen monitor located at approximately 60 cm from the subject. Participants were randomly assigned to six different picture presentation sequences that followed a set of eight 3×3 Latin squares (72 trials, 6 trials per picture) to guarantee that all pictures had an equal preference distribution. Each presentation consisted of a 4-sec baseline, 6-sec picture presentation, and 4-sec post-picture interval. Two-thirds of the trials (48), equally distributed across the three face categories, were presented together with a startle probe (a burst of white noise at 105 dBs, 50-ms duration and nearly instantaneous rise time) at 4, 4.5, 5 or 5.5 sec after picture onset. Duration of the physiological test was around 30 minutes.

### Physiological Measures

Left orbicularis and zygomatic EMG activity were measured using Coulbourn V75-04 bioamplifiers and V76-24 integrators. Time constants and sampling rates for zygomatic and orbicularis muscles were 500 and 20 ms, and 100 and 1000 Hz, respectively. Heart rate was derived from the electrocardiogram recorded with a V75-04 bioamplifier at lead II and sampled at 1000 Hz. Skin conductance was recorded using a V75-23 bioamplifier with the electrodes placed on the hypothenar eminence of the left hand. The signal was acquired at a sampling rate of 50 Hz.

### Self-report Measures

The startle probe paradigm uses three pictographic scales entitled the Self-Assessment Manikin [32] to assess three bipolar emotional dimensions: Valence (pleasant-unpleasant), Arousal (relaxed-activated), and Dominance (dominant-dominated). Each scale depicts five humanoid figures (from a sad to a happy face for valence, from a relaxed to an exited body for arousal, and from a very small to a very large body for dominance) that represent the intensity levels of each dimension providing a score that ranges from 1 to 9.

### Procedure

We first contacted participants by phone to invite them to attend two laboratory sessions. The first session ensured that participants complied with the inclusion criteria. They completed the questionnaires mentioned above and were provided with the camera and instructions on how to take the photographs. At the second session, we administered the physiological test to participants. Upon arrival in the laboratory, we invited the participant to sit on a reclining chair in a dimly lit room. After we placed the sensors, participants viewed the pictures as explained above. We instructed them to view each picture for the entire time it was on screen. After this task, we removed the sensors, and the participant evaluated the valence, arousal, and dominance of the 12 pictures using the Self-Assessment Manikin. Finally, we thanked participants for their time and fully explained the purpose of our study.

### Data Reduction and Analysis

The startle reflex amplitude was defined as the difference in microvolts between the peak and the onset of the response, in a time window between 20 and 120 ms after stimulus onset, scored by means of the algorithm described by Balaban et al. [38]. To control for between-subject variability, startle amplitude for each subject was converted to standardized *t* scores. Responses in heart rate, skin conductance, and zygomatic EMG activity were determined by averaging across each half-second during the 6-sec picture presentation and subtracting that activity from the activity obtained 3 seconds before picture onset. Data analysis for eye-blink startle and subjective measures was performed using ANOVAs, with Gender as a between-subjects factor and Face Category as a repeated-measures factor. For zygomatic activity, heart rate, and skin conductance, a second repeated-measures factor of Time was added (the 12 half-second bins through the duration of the picture display). The Greenhouse-Geisser correction was used to correct any violation of sphericity in the repeated-measures factors. Post-hoc planned comparisons between loved, neutral, and unpleasant faces were conducted using Bonferroni test. The level of significance was set at 0.05 for all analyses.

## Results

### Loved versus Neutral versus Unpleasant Faces

#### Startle reflex

**Figure 1:**
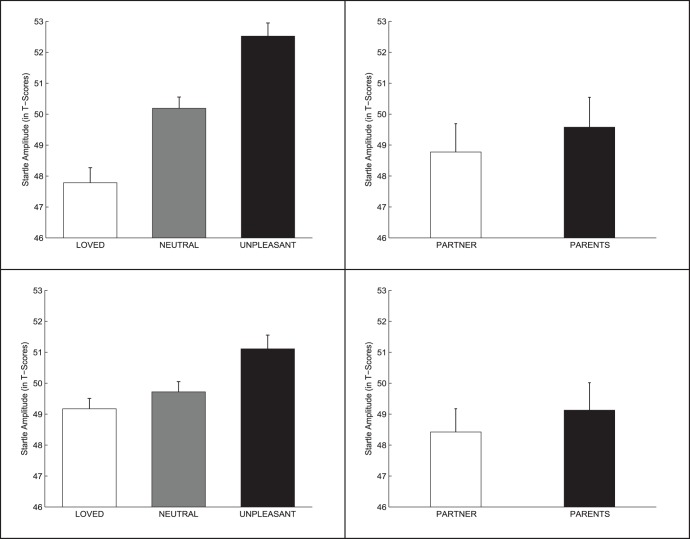
Startle reflex response to the faces. Magnitude of the startle reflex to the acoustic noise while participants viewed faces. Left: Loved vs. neutral vs. unpleasant faces (top: females; bottom: males). Right: Romantic partner (boyfriend/girlfriend) vs. same-sex parent (father/mother) faces (top: females; bottom: males). Bars are standard errors.

The 2 (Gender) ×3 (Face Category) ANOVA results yielded a significant effect of Face Category (F (2, 104) = 24.11, *p*<0.0001; ηp^2^ = 0.317) and a significant Face Category × Gender interaction (2, 104) = 4.38, *p*<0.02 ηp^2^ = 0.078). No significant main effect of Gender was found. Figure 1 (left panel) shows the magnitude of the average eye-blink startle to the acoustic sound when female (top) and male (bottom) participants were viewing each of the three face categories. For both gender groups, the startle reflex showed reduced amplitude while viewing loved faces and increased amplitude when viewing unpleasant faces, compared to neutral ones. However, the differences were larger for females than for male participants. Analysis of the interaction showed a significant linear trend in both groups (females: *p*<0.0001; males: *p*<0.012), but the slope of the trend was significantly larger in the female group (*p*<0.009). In this group, startle magnitude while viewing loved faces was significantly reduced compared to both neutral (*p*<0.007) and unpleasant (*p*<0.0001) faces. In the male group, significant differences were limited to the comparison between loved and unpleasant faces (*p*<0.04).

#### Zygomatic muscle activity

**Figure 2:**
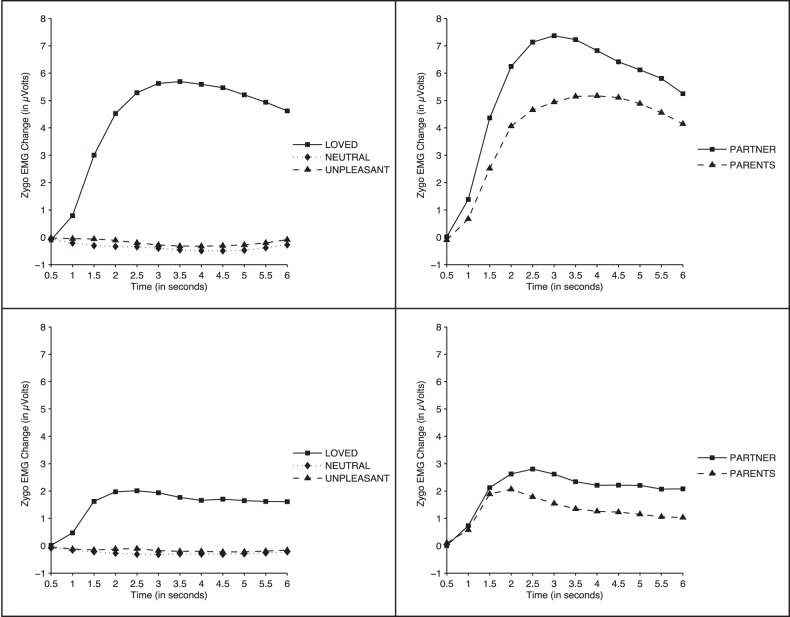
Zygomatic muscle response to the faces. Zygomatic muscle activity while participants viewed faces. Left: Loved vs. neutral vs. unpleasant faces (top: females; bottom: males). Right: Romantic partner (boyfriend/girlfriend) vs. same-sex parent (father/mother) faces (top: females; bottom: males).

The 2 (Gender) ×3 (Face Category) ×12 (Time) ANOVA results yielded significant effects for Face Category (F (2, 104) = 19.69, *p*<0.0001, ηp^2^ = 0.275), Time (F (11, 572) = 13.81, *p*<0.0001, ηp^2^ = 0.210 ), Gender (F (1, 52) = 4.92, *p*<0.03, ηp^2^ = 0.087), Face Category × Time (F (22, 880) = 17.80, *p*<0.0001, ηp^2^ = 0.255), Gender × Time (F (11, 572) =  *p*<0.02, ηp^2^ = 0.084), and Gender × Face Category × Time (F (22, 1144) = 5.29, *p*<0.02, ηp^2^ = 0.084). Figure 2 (left panels) shows changes in zygomatic muscle activity during picture presentation for both female (top) and male (bottom) participants. In both groups, loved, familiar faces prompted a clear response starting almost immediately after the picture presentation onset and continuing until the offset of the image. The response was significantly larger in women than in men, and in both groups it was significantly larger to loved faces than to neutral and unpleasant faces from second 1.5 through the offset of picture presentation (all *p*-values <0.03 for women, and <0.05 for men). No significant differences were found between neutral and unpleasant faces (*p*>0.18 for women, and *p*>0.6 for men).

#### Heart-rate

**Figure 3:**
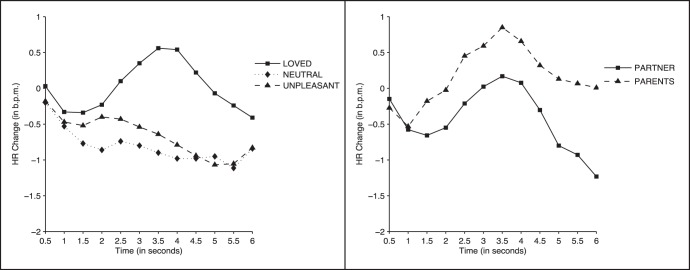
Heart rate response to the faces. Heart-rate changes while participants viewed faces. Left: Loved vs. neutral vs. unpleasant faces. Right: Romantic partner (boyfriend/girlfriend) vs. same-sex parent (father/mother) faces.

The 2 (Gender) ×3 (Face Category) ×12 (Time) ANOVA results yielded significant effects for Time (F (11, 572) = 4.78, *p*<0.003; ηp^2^ = 0.084) and Face Category × Time (F (22, 1144) = 3.64, *p*<0.004; ηp^2^ = 0.065). No significant effect of Gender was found. Figure 3 (left panel) displays the heart-rate response during picture presentation for all three face categories. Neutral and unpleasant faces induced a decelerative response that was maintained throughout the entire period of picture presentation. In contrast, loved faces, after an *initial* deceleration, induced a cardiac acceleration between seconds 2,5 and 5, with a peak at 3.5 seconds. Significant differences between loved and neutral faces were found at all time points between seconds 2 and 5.5 (all *p*-values <0.04). Significant differences between loved and unpleasant faces were found between seconds 3 and 5 (all *p*-values <0.04). No significant differences were found between unpleasant and neutral faces (*p*>0.6).

#### Skin conductance

**Figure 4:**
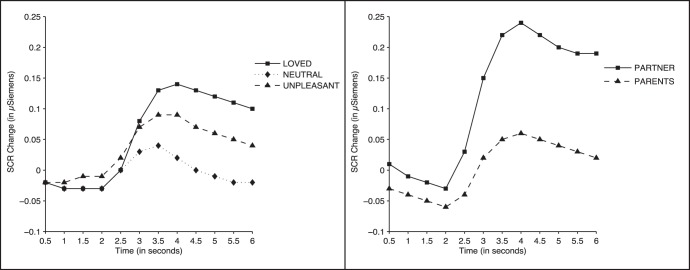
Skin conductance response to the faces. Skin conductance changes while participants viewed faces. Left: Loved vs. neutral vs. unpleasant faces. Right: Romantic partner (boyfriend/girlfriend) vs. same-sex parent (father/mot her) faces.

The 2 (Gender) ×3 (Face Category) ×12 (Time) ANOVA results showed significant effects of Face Category (F (2, 104) = 7.11, *p*<0.001, ηp^2^ = 0.120), Time (F (11, 572) = 27.34, *p*<0.0001, ηp^2^ = 0.345), and Face Category × Time (F (22, 1144) = 8.34, *p*<0.0001, ηp^2^ = 0.138). No significant effect of Gender was found. Figure 4 (left panel) shows the skin conductance response. All picture categories produced a response starting approximately 2.5 seconds after picture onset. Responses to loved and unpleasant faces were significantly larger than responses to neutral faces at all time points between seconds 4 and 6 (all *p-*values <0.04). No significant differences were found between loved and unpleasant faces (all *p-*values >0.16).

#### Subjective ratings

Table 1 shows the mean and standard deviation scores for the participants’ self-report ratings of valence, arousal, and dominance. The 2 (Gender) ×3 (Face Category) ANOVAs yielded significant main effects for all three scales, Valence (F (2, 104) = 409. 07, *p*<0.0001; ηp^2^ = .887), Arousal (F (2, 104) = 41.33, *p*<0.0001; ηp^2^ = 0.443), and Dominance (F (2, 104) = 24.34, *p*<0.0001; ηp^2^ = 0.319), and significant interactions of Valence × Gender (F (2, 104) = 6.54, *p*<0.006; ηp^2^ = 0.112) and Arousal × Gender (F (2, 104) = 6.02, *p*<0.004; ηp^2^ = 0.104). For the valence scale, in both men and women, viewing loved faces elicited higher pleasant feelings than viewing neutral and unpleasant faces (all *p*-values <0.0001), the pleasant feelings for loved faces being higher in women than men (*p*<0.0001); for the arousal scale, in both genders, viewing loved faces elicited higher feelings of arousal than neutral faces (*p*<0.02), but lower feelings of arousal than unpleasant faces, the arousal feelings of unpleasant faces being higher for women than men (*p*<0.05) and the arousal feelings of pleasant and neutral faces being higher for men than women (both *p*-values <0.04); and for the dominance scale, viewing loved faces elicited higher feelings of dominance than unpleasant faces (*p*<0.0001) but no significant differences with respect to neutral faces (*p*>0.5).

**Table 1:** Subjective ratings of Valence, Arousal, and Dominance for the faces.

	Women (N = 30)			Men (N = 24)		
Pictures	Valence	Arousal	Dominance	Valence	Arousal	Dominance
Loved	8.5 (0.7)	3.9 (2.5)	5.1 (1.5)	7.6 (0.8)	5.3 (2.1)	5.6 (1.4)
Neutral	4.9 (0.9)	2.7 (1.6)	5.6 (1.2)	4.8 (0.4)	3.7 (1.4)	5.7 (1.2)
Unpleasant	2.0 (1.4)	6.7 (1.6)	4.2 (1.3)	2.7 (1.2)	5.8 (1.5)	4.2 (1.1)
Partner	8.8 (0.6)	4.5 (2.9)	5.3 (2.0)	8.2 (1.0)	6.0 (2.6)	6.5 (1.4)
Parent	8.3 (0.9)	3.7 (2.6)	5.0 (1.7)	7.5 (1.4)	5.0 (2.3)	5.3 (2.2)

Mean (standard deviation) of subjective ratings of Valence, Arousal, and Dominance for loved, neutral, unpleasant, partner, and parent faces reported by women and men (score range: 1–9).

### Romantic Partner versus Father/Mother Face

#### Startle reflex

Figure 1 (right panel) shows the magnitude of the average eye-blink startle to the acoustic sound when female (top) and male (bottom) participants were viewing the faces of the romantic partner (boyfriend or girlfriend) and the parent of the same gender as the partner (father or mother). The 2 (Gender) ×2 (Face Category) ANOVA yielded no significant effects (all *p*-values >0.43. The startle response was not significantly different across face categories.

#### Zygomatic muscle activity

Figure 2 (right panel) shows the zygomatic muscle response elicited by the faces of the romantic partner and the face of the parent of same gender. The 2 (Gender) ×2 (Face Category) ×12 (Time) ANOVA yielded significant effects of Gender (F (1, 52) = 4.53, *p*<0.003, ηp^2^ = 0.162), Face Category (F (1, 52) = 10.90, *p*<0.002, ηp^2^ = 0.173), Time (F (11, 572) = 15.37, *p*<0.0001, ηp^2^ = 0.228), and Gender × Time (F (11, 572) = 5.14, *p*<0.01, ηp^2^ = 0.090). As illustrated in Figure 2, the face of the romantic partner showed a larger zygomatic response than the father/mother face in both male and female participants (*p*<0.002), but the two responses of female participants were significantly larger than the two responses of male participants from second 3 to second 6 (all *p*-values <0.04).

#### Heart rate

Figure 3 (right panel) shows the heart rate response when participants were viewing the faces of the romantic partner and the face of the parent of same gender. The ANOVA results yielded only a significant effect of time (F (11, 572) = 3.92, *p*<0.009, ηp^2^ = 0.070). Face Category and Gender showed no significant effects.

#### Skin conductance

Figure 4 (right panel) shows the skin conductance response when participants were viewing the faces of the romantic partner and the face of the parent of same gender. The ANOVA results yielded significant effects of Time (F (11, 572) = 26.00, *p*<0.0001, ηp^2^ = 0.333), Face Category (F (1, 52) = 9.90, *p*<0.003, ηp^2^ = 0.160), and Face Category × Time (F (11, 572) = 8.61, *p*<0.0001, ηp^2^ = 0.142). No gender effect was found. As illustrated in Figure 4, the face of the romantic partner showed a significantly larger response than the face of the father/mother from second 3 to second 6 (all *p*-values <0.008).

#### Subjective ratings

Table 1 shows the mean (and standard deviation) scores for the participants’ subjective ratings of valence, arousal, and dominance in response to the romantic partner and same-gender parent faces. Participants rated the romantic partner’s face as eliciting higher feelings of pleasantness, arousal, and dominance than the same-gender parent’s face (valence (F (1, 52) = 11.90, *p*<0.001; ηp^2^ = .186); arousal (F (1, 52) = 11. 02, *p*<0.002; ηp^2^ = .175); dominance (F (1, 52) = 6. 66, *p*<0.02; ηp^2^ = .113)). Significant main effects of gender were also found for the valence and arousal scales. Women rated their father’s and romantic partner’s faces as eliciting higher feelings of pleasantness than men (F (1, 52) = 9.76, *p*<0.003; ηp^2^ = .158), but men rated both faces as eliciting higher feelings of arousal than women (F (1, 52) = 4.29, *p*<0.04; ηp^2^ = .076).

## Discussion

These results indicate that, for both men and women, viewing loved, familiar faces inhibits paradigmatic defense reactions, such as the eye-blink startle reflex. They also replicate previous findings of peripheral electrophysiological responses shown by women in reaction to loved, familiar faces [30], [31] and extend the same findings to men. Nevertheless, there were gender differences in terms of the magnitude of some physiological and subjective responses. Women showed a larger startle inhibition and a larger zygomatic response to loved faces than men, accompanied by higher ratings of positive feelings but lower ratings of arousal. In general, our results reinforce the interpretation of the physiological and subjective responses to loved, familiar faces as elicitation of an intense and positive emotional response that is not attributable to undifferentiated arousal or familiarity. Here we discuss the implications of our findings regarding the startle reflex inhibition, the arousal and familiarity issues, and the potential brain mechanisms linking loved faces to health benefits.

To date, the augmentation and inhibition of the startle reflex by viewing emotional pictures has only been consistently demonstrated through use of complex scenes from the *International Affective Picture System* (IAPS) [32] contrasting highly unpleasant (e.g., threatening people or phobic animals) with highly pleasant (e.g., erotic couples or sport images) content. Blink modulation using simple pictures, such as faces showing emotional expressions (e.g., happy, fearful, or angry faces), has remained elusive [39], [40]. Previous studies on adults have reported either no effect of facial expression on startle modification [41], blink potentiation only to male actors displaying negative emotions [42], or startle potentiation only to fearful and angry facial expressions [39], [40]. To date, no study has reported startle inhibition by affective faces. In the context of these null results, our finding of a marked inhibition of the eye-blink startle reflex in response to viewing loved, familiar faces, highlights the capacity of the faces of loved ones to inhibit defense reactions, even when the faces are presented as black-and-white photographs devoid of emotional expression.

This inhibitory capacity is likely to be rooted in biology. The face represents a key aspect of social and emotional communication. It conveys information about the feelings and identity of people, which are two essential cues that help discriminate friendly (i.e., social inclusion) and hostile (i.e., social exclusion) attitudes and intentions. Given such relevance, it is no surprise that the face had been the subject of much research in the past. Although most studies have followed Darwin’s seminal work, outlined in *The expression of emotions in man and animals,* and focused on emotional facial expressions, a number of recent studies have specifically examined the electrophysiological (ERP) and hemodynamic (fMRI) indices of brain activity associated with the recognition of loved, familiar faces [16]–[24]. As noted in the introduction, one major limitation of these studies is the lack of control for undifferentiated emotional arousal and familiarity. None of these previous studies used peripheral physiological indices to distinguish positive affect from overall arousal and familiarity. Most prior studies relied on subjective reports, a method with obvious validity problems [43]. On the other hand, previous research attempted to control for familiarity by including the faces of acquaintances, famous people, friends, or newly learned faces. However, the familiarity of loved people will always exceed that of the control faces because of the greater amount of knowledge about and time spent with loved ones [23].

Our physiological results using Lang’s *startle probe paradigm* confirm that viewing loved faces elicits an intense and positive emotional response that is not due to undifferentiated emotional arousal. Zygomatic and heart-rate responses (two specific indices of positive emotion) were larger in response to loved faces than to neutral or unpleasant faces. Skin conductance (a specific index of emotional arousal), as expected, was larger in response to both loved and unpleasant faces than to neutral faces. Thus, physiological measures confirm subjective ratings in indicating the presence of both positive valence and intense arousal in response to loved faces. Although similar responses were found across female and male participants, we observed gender differences in response to loved faces. The magnitude of the zygomatic response and valence ratings were greater in female than male participants, whereas arousal ratings were greater in male than in female participants. These differences are consistent with reports of women’s greater zygomatic activity when viewing happy faces [44] and IAPS pictures of families [45]. They are also consistent with our finding of greater startle inhibition in women while viewing loved faces. According to the *motivational priming hypothesis*, the greater the activation of the appetitive motivational system by the pleasant stimuli, the greater the magnitude of startle inhibition [26].

Physiological and subjective results when comparing loved faces with different levels of familiarity (romantic partner and father/mother of same gender as partner) confirm that familiarity is not the key factor in explaining the observed physiological responses. In the context of research on recognition of familiar faces, familiarity refers to factual knowledge about the person being recognized and has been operationalized in terms of amount of time spent with the person [23]. In our study, all responses showed similar (heart-rate) or larger (zygomatic activity and skin conductance) responses to the less familiar face (the romantic partner), in conjunction with higher ratings of pleasantness, arousal, and dominance. The larger responses to the romantic partner can be explained by the presumably higher positive emotionality present in romantic love, due to the presence of sexual attraction, a love component absent in filial love [5], [31]. Thus our results, which replicate previous findings in female students comparing filial versus romantic love [31], reinforce the interpretation of the observed physiological responses to loved faces as due to the higher subjective evaluations of the faces (higher valence and arousal) rather than to differences in familiarity. On the other hand, the higher rating of dominance to the romantic partner, compared to the father/mother of same gender, which also replicates previous findings [30], [31], reinforce the interpretation of the dominance scale in terms of protection or control: participants feel more protected or controlled (feel small) when viewing the face of the father or mother than when viewing the face of the romantic partner.

The relevance of our findings should be evaluated taking into consideration some methodological limitations. Gender differences in our study should be taken cautiously since male and female participants were not balanced in our sample. Moreover, participants were all university students and, consequently, extension of our findings to other populations is not warranted. Keeping these limitations in mind, the finding that viewing loved faces inhibits the startle reflex, together with evidence that such inhibition is accompanied by subjective and physiological responses that indicate the presence of an intense, positive emotional response, supports the hypothesis that loved faces may function as safety cues that activate the appetitive reward system and reciprocally inhibit defense reactions. The neural mechanisms underlying this reciprocal inhibition are still not well understood. However, based on neuroimaging studies of the brain areas activated by loved faces [14], [16], [17], [21], [24], together with data on the brain mechanisms that modulate the startle reflex [46], [47] and pain responses [14], we may speculate that such mechanisms involve, in addition to activation of the reward system, activation of prefrontal areas known to exert an inhibitory role on subcortical structures, such as the amygdala, which directly modulate the startle reflex and other defense reactions [46], [47]. Inhibition of defense reactions, with their broad spectrum of physiological and endocrine stress responses, may contribute in the long term to the positive health outcomes consistently reported in the scientific literature associated with social support.

In summary, the present study shows that viewing loved, familiar faces inhibits the eye-blink startle reflex. Additionally, it replicates previous findings in women regarding greater physiological and subjective responses to loved faces, and extends the same findings to men. This set of data highlights the capacity of loved faces to elicit an intense positive emotional response and simultaneously inhibit defense reactions. We conclude that this inhibitory capacity may contribute to the health benefits associated with social support.
